# Development of Monitoring System for Assessing Rheumatoid Arthritis within 5 Minutes Using a Drop of Bio-Fluids

**DOI:** 10.3390/jcm9113499

**Published:** 2020-10-29

**Authors:** Jung Hee Koh, Saseong Lee, Hyun-Sook Kim, Kyuheon Lee, Chang Seop Lee, Seung-Ah Yoo, Naeun Lee, Wan-Uk Kim

**Affiliations:** 1 Division of Rheumatology, Department of Internal Medicine, The Catholic University of Korea, Seoul 06591, Korea; jungheekoh@catholic.ac.kr; 2 Center for Integrative Rheumatoid Transcriptomics and Dynamics, The Catholic University of Korea, Seoul 06591, Korea; l221@naver.com (S.L.); youcap78@hanmail.net (S.-A.Y.); nelee2015@catholic.ac.kr (N.L.); 3 Department of Internal Medicine, Soonchunhyang University College of Medicine, Seoul 04401, Korea; healthyra@sch.ac.kr; 4 NanoEntek Inc., Seoul 08389, Korea; khlee@nanoentek.com (K.L.); cslee@nanoentek.com (C.S.L.); 5 Department of Biomedicine & Health Sciences, The Catholic University of Korea, Seoul 06591, Korea

**Keywords:** rapid quantitative immunoassays, FREND™ system, soluble CD14, rheumatoid arthritis, biomarker

## Abstract

Rheumatoid arthritis (RA) disease activity fluctuates over time. The disease activity score 28 (DAS28_ESR_) is a widely used and validated scoring system for assessing RA activity; however, it requires time and expertise. This study aimed to develop a new molecular assay capable of rapidly and objectively assessing RA activity. We used a rapid immuno-assay system (FREND™) to measure soluble CD14 (sCD14) levels, which reflect the DAS28_ESR_. SCD14 concentrations in urine and serum of RA patients were measured, and RA activity and responses to anti-rheumatic drugs were examined at baseline and after 6 months. FREND™ quantified sCD14 levels in a drop of serum and urine accurately and within 5 min. Serum sCD14 concentrations and its changes correlated well with disease activity and treatment responses, and the results were comparable to C-reactive protein. The new composite indices, including the DAS28_CD14_ and simplified DAS_CD14_, better detected RA activity than a single sCD14 value and correlated strongly with the DAS28_ESR_. These indices exhibited excellent diagnostic performance for discriminating a good response 6 months after treatment. We developed a new system for assessing RA activity and therapeutic outcome within 5 min. CD14-based composite indices may have utility for accurate and frequent monitoring of RA status.

## 1. Introduction

The treat-to-target strategy has improved the prognosis of rheumatoid arthritis (RA) greatly [1]. The target is to achieve remission, or low disease activity if remission is not possible, particularly in those with long-standing disease [2]. To this end, the disease activity score 28 (DAS28) was developed and is used widely to assess RA activity [2]. However, the index is intricate because it comprises several parameters, including the number of tender and swollen joints and a global assessment (by the patient and or caregiver), and requires considerable time and expertise. Thus, there is an unmet need for more simple biomarkers that accurately reflect RA activity and predict responses to anti-rheumatic drugs. The erythrocyte sedimentation rate (ESR) and C-reactive protein (CRP) level are blood biomarkers used to monitor RA activity; however, they have limited specificity and/or sensitivity [3,4]. Moreover, emerging evidence suggests that CRP may not be a reliable biomarker in patients treated with biologics, particularly anti-IL-6 blocking agents [5]. Thus, it is important to identify alternative and reliable biomarkers that can improve assessment of the RA disease activity.

Molecular approaches have also been used to assess RA activity; these include a multi-biomarker disease activity (MBDA) score based on the concentrations of twelve proteins [6]. The MBDA is more accurate to some extent, but it takes considerable time to get the test results and it is expensive. Moreover, some reports demonstrate that it is of little (if any) value for assessing RA activity, or for predicting the risk of radiographic progression or flare-ups [7,8,9]. Therefore, cost-benefit analysis suggests that the MBDA may not be a suitable alternative to the therapeutic decision-making process. Recently, we undertook transcriptomics and proteomics studies that identified three molecular candidates in urine (orsomucoid (ORM)1, ORM2, and soluble CD14 (sCD14)) that reflect RA activity [10,11]. In particular, sCD14, a protein primarily produced by monocytes/macrophages and hepatocytes, has diagnostic value comparable with that of conventional serum markers, and it has even greater predictive power for disease activity when combined with serum CRP [11,12], suggesting that sCD14 identifies a diagnostic window complementary to that of current serum parameters for disease activity.

RA disease activity fluctuates over time. Cumulative disease activity is the major determinant of joint damage and extra-articular manifestations of RA [13]. In this sense, stable maintenance of remission or low disease activity, suggested by the target-to-treat strategy, seems to be critical for achieving therapeutic goals [1,2]. Although it is unclear how many times per year the DAS28 should be measured, it may be ideal (theoretically) to monitor RA activity on a regular basis. One of the ways to address this would be to develop a rapid detection system that patients can use to reliably and accurately self-monitor a simple biomarker that represents RA activity, much as we do for blood pressure and glucose measurement. Since cumulative or multiple test results more accurately reflect RA activity than single on-visit data [13], such a self-monitoring system may help rheumatologists to better control RA activity and prevent complications.

For most clinicians, having immediate test results, like blood glucose levels, is of great help to the decision-making process, particularly when titrating medications. To achieve this goal, trials of a new immunoassay system using micro-fluidics and nanotechnology to rapidly measure levels of a certain protein in bio-fluids have been conducted. Here, we report a new assay system capable of accurately quantifying sCD14 levels in a drop of serum and urine within 5 min. Using this system, we investigated whether sCD14 levels reflect disease activity and treatment responses in RA patients.

## 2. Materials and Methods

### 2.1. Study Population

The Center for Integrative Rheumatoid Transcriptomics and Dynamics (CIRAD) cohort is a prospective cohort of RA patients at Seoul St. Mary’s Hospital, the Catholic University of Korea. The CIRAD cohort, formed in 2015, has obtained overall clinical data and samples from RA patients every 6 months. All patients fulfilled the 2010 from American College of Rheumatology (ACR)/European League Against Rheumatism (EULAR) RA classification criteria [14]. This study was approved by the institutional review board of Seoul St. Mary’s Hospital, the Catholic University of Korea (KC16SISI0632). Written informed consent was obtained from all study participants.

### 2.2. Assessment of Clinical Parameters

Clinical variables include swollen joint count (SJC), tender joint count (TJC) (including 28 joints), patient global assessment of disease activity (PGA) (0–100 mm, 100 = the worst score), ESR (mm/h), and CRP (mg/dL). The DAS28_ESR_ was calculated based on the SJC, TJC, ESR, and PGA. Disease activity was assessed by trained investigators at every evaluation using the DAS28_ESR_. Disease activity was categorized as low (DAS28_ESR_ ≤ 3.2), moderate (3.2 < DAS28_ESR_ ≤ 5.1), or high (DAS28_ESR_ > 5.1) based on ACR recommendations [15]. The effectiveness of treatment was assessed according to EULAR treatment responses using the DAS28_ESR_ [16,17]. The presence or absence of comorbidities was recorded by investigators at interview. The case report forms did not specify diagnostic or classification criteria for diseases other than RA.

**Figure A1:**
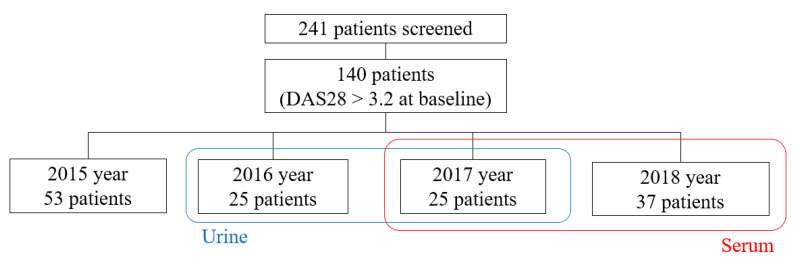
Patients in the CIRAD cohort. Patients were selected from the Center for Integrative Rheumatoid Transcriptomics and Dynamics (CIRAD) cohort. Enrollment in the cohort took place from 2015 and collection of follow-up data is still ongoing. This research and development progressed in stages. In the first stage, validation of sCD14 concentration using the FREND™ system was performed using urine samples, which were collected from 2016 to 2017. In the second stage, validation was performed using serum samples collected from 2017 to 2018.

By December 2018, 319 patients were enrolled in the CIRAD cohort and 241 completed a 6-month visit. To investigate changes of sCD14 in serum and urine according to treatment response, 140 patients with moderate-to-high disease activity (DAS28_ESR_ > 3.2) at baseline were selected (Figure A1 and Table A1). To validate the accuracy of a new diagnostic system (FREND™-sCD14 System), 100 urine samples from 50 patients enrolled from 2016 to 2017 were used (Study I). To determine whether serum sCD14 levels represent treatment response to anti-rheumatic drugs, 124 serum samples from 62 patients enrolled from 2016 to 2018 were used (Study II).

### 2.3. Collection of Serum and Urine

Serum and urine samples were obtained at routine outpatient clinics. Blood and urine samples were collected in plain tubes and centrifuged at 1008× *g* for 10 min. Next, serum and clarified urine supernatant were collected and frozen at −70 °C until use.

### 2.4. Validation of the sCD14 Test Cartridge

Levels of sCD14 in the paired urine samples were measured by enzyme-linked immunosorbent assay (ELISA) and then compared with test results from the FREND™-CD14 system (at http://www.nanoentek.com). The human CD14 ELISA kit (R&D Systems, Minneapolis, MN, USA) was used according to the manufacturer’s instructions. Levels of sCD14 in the same sample were measured in duplicate in both the ELISA and FREND™ system.

### 2.5. Statistical Analysis

Continuous variables were compared using the Mann–Whitney U test. Categorical variables were compared using the Chi-Square test or Fisher’s exact test as appropriate. Correlations between two variables were analyzed using Spearman’s correlation test. A Wilcoxon’s signed-rank test was employed to examine differences between measurements at baseline and the 6-month visit. Multivariate analysis of covariance (ANCOVA) was performed to determine whether the simplified DAS_CD14_ and DAS28_CD14_ scores were independent predictors of disease activity after adjusting for age, sex, serologic profile, comorbidities, and medications.

Cronbach’s α was calculated to assess the reliability of sCD14 levels measured by the sCD14 test cartridge. Receiver operating characteristics (ROC) curves were drawn to determine the predictive value of moderate-to-high disease activity (DAS28_ESR_ > 3.2) and the good response according to EULAR. The area under curve (AUC) of sCD14-based disease activity scores and CRP-based disease activity scores measured in the same subject were compared by mathematical equivalence using the Mann–Whitney U statistic [18]. Agreement between the classification of disease activity (low, moderate, and high disease activity) according to the simplified DAS_CD14_ and according to DAS28_ESR_ was assessed by calculating Cohen’s κ. Statistical analyses were conducted using SAS version 9.4 (SAS Institute, Cary, NC, USA) and graphs were drawn using GraphPad Prism 8 (GraphPad Software, San Diego, CA, USA).

## 3. Results

### 3.1. Development of the FREND System for Rapid Detection of sCD14 in Bio-Fluids

**Figure 1:**
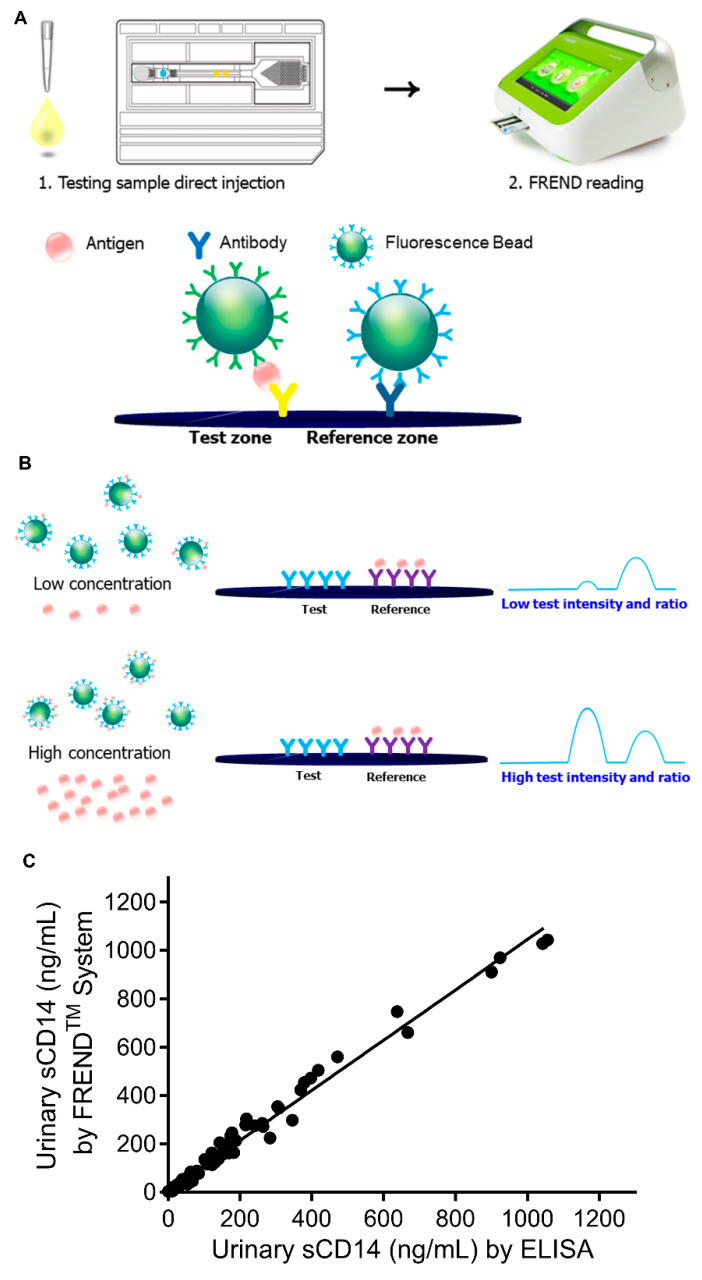
Summary and explanation of the test. (**A**) To perform the test, a 35 μL sample is added to the single-use FREND™-CD14 cartridge, where it is mixed with fluorescent nanoparticles conjugated to anti-CD14 antibodies. The FREND™-CD14 system includes a bench-top fluorescence reader [240 mm (width) × 260 mm (length) × 175 mm (height)] with a slot that accepts the FREND™-CD14 cartridge. Molecules in the specimen bind to the conjugated anti-CD14 antibodies to form immune complexes, which then move by capillary action through the reagent cartridge channel to the detection area. When the specimen reaches the test zone, it hydrates the dried solid-phase anti-sCD14 antibodies. (**B**) The result is based on the ratio of fluorescence in the FREND™-CD14 cartridge test and reference zones. The magnitude of the fluorescent ratio is inversely proportional to the amount of sCD14 in the sample. (**C**) The validity of the FREND™-CD14 system for measuring sCD14 concentrations.

The FREND™ system is a portable cartridge reader that measures laser-induced fluorescence in a single-use reagent cartridge [19]. To develop a rapid quantitative measurement system for sCD14 (hereafter termed the ‘FREND™-CD14 system’), we designed a portable fluorescence reader with a slot that accepts the sCD14 test cartridge (Figure 1A). The instrument includes a touchscreen interface and is programmed to interpret the test after a sample has reacted completely with the on-board cartridge reagents. Test results are displayed on the screen and transferred to a mobile application on a cell phone and can then be transported to physicians’ personal computers at the outpatient clinic. The cartridge encloses a rapid quantitative sandwich immunoassay based on fluorescent nanoparticles, which measures the concentration of sCD14 (Figure 1A).

To measure sCD14 levels using the FREND™-CD14 system, a single drop (35 μL) of serum or urine is added to a dilution solution and then applied to the inlet of the cartridge, where it is mixed with fluorescent nanoparticles conjugated to anti-CD14 Abs; when the specimen reaches the test zone, it hydrates the dried anti-CD14 Abs on the solid-phase (Figure 1A). The reaction and analysis time are approximately 3 min for the CD14 test cartridge and 1 min for the cartridge reader of FREND™-CD14 system. The sCD14 concentration is calculated based on the ratio of the fluorescence at the test and reference zones within the CD14 cartridge; the magnitude of the fluorescent ratio is inversely proportional to the amount of sCD14 in the sample. Thus, a lower fluorescence ratio correlates with a higher concentration of sCD14 (Figure 1B). The sCD14 test cartridge has a limit of blank value of 0.01 ng/mL, a limit of detection value of 0.04 ng/mL, and a limit of quantitation value of 0.5 ng/mL. It meets the acceptance criteria for inter-assay precision tests at three different concentrations (Supplemental Table S1). The recovery of sCD14 from a sample containing various potential interfering substances, including acetylsalicylic acid, prednisolone, methotrexate, and hydroxychloroquine, is also within acceptable ranges (Supplemental Table S2).

### 3.2. Validity and Reliability of FRENDTM-CD14 System

To validate the FREND™-CD14 system, 100 paired urine samples from 50 RA patients were analyzed. The baseline characteristics of the patients are described in Figure A1 and Table A1 (study I). As a standard for comparison with the FREND™-CD14 system, a conventional solid phased ELISA for sCD14 was performed using the same urine samples. As seen in Figure 1C, the values using the two methods showed an excellent correlation (Spearman’s *rho* = 0.993, *p* < 0.001). To assess reliability, the sCD14 concentration in each sample was measured twice (at different time intervals) using the FREND™-CD14 system, which revealed high internal consistency: Cronbach’s α = 0.996 (95% CI, 0.994–0.997). These results indicate that the FREND™-CD14 system shows excellent analytic performance for determination of sCD14 levels, and that the time required to obtain a result was much shorter than that for the conventional ELISA system (< 5 min versus > 6 h, respectively).

### 3.3. The FREND™-CD14 System Shows Weak Diagnostic Performance When Testing Urinary sCD14

**Figure A2:**
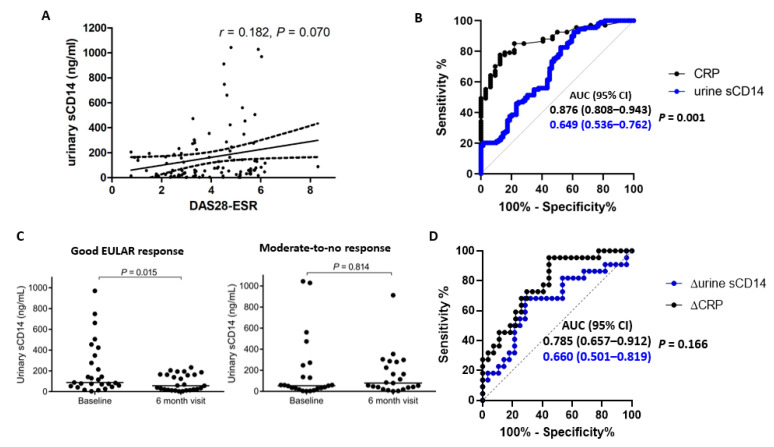
Comparison of urinary sCD14 concentrations measured by the FREND™ system with RA disease activity and treatment outcome. (**A**) Linear correlation between urinary sCD14 and the DAS28_ESR_. (**B**) ROC curve analysis of urinary sCD14 versus CRP levels for differentiating low RA activity from moderate-to-high RA activity. Two AUCs were compared using two-sided statistical tests and *p*-values were calculated. (**C**) Urinary sCD14 levels according to EULAR treatment response, and (**D**) ROC curve analysis of Δurinary sCD14 (sCD14 level at baseline to the sCD14 level at 6 months after treatment with anti-rheumatic drugs) for differentiating good responders from moderate-to-no responders. The AUC value for changes in CRP (ΔCRP) is shown for comparison. Two AUCs were compared, and the *p*-value does not indicate a significant difference between them.

Urine is more stable than other bio-fluids [20] and can be a rich source of biomarkers reflecting systemic inflammation [21]. Recently, we demonstrated that urinary sCD14 concentrations measured by ELISA are representative of RA activity; they correlate, albeit modestly, with conventional inflammatory markers in blood (including ESR and CRP) and the DAS28 [22]. DAS stands for ‘disease activity score’ and the number 28 indicates the 28 joints subjected to assessment [22]. The DAS28 can include ESR or CRP, known as the DAS28_ESR_ or DAS28_CRP_, respectively. To investigate whether sCD14 levels measured using the FREND™-CD14 system reflect RA activity, the data (n = 100 samples described above) were compared with DAS28 values assessed simultaneously at the time of sampling. We found that urinary sCD14 levels and the sCD14-to-creatinine ratio in urine tended to correlate with the DAS28_ESR_, although the data did not reach statistical significance (*rho* = 0.182 (*p* = 0.070) and 0.188 (*p* = 0.066), respectively) (Figure A2A). Urinary sCD14 concentrations distinguished patients with low disease activity (DAS28_ESR_ ≤ 3.2) from those with moderate-to-high disease activity (DAS28_ESR_ >3.2) [15,22], but the discriminative power was not strong (AUC = 0.648, *p* = 0.021) (Figure A2B).

Of the 50 RA patients from whom serial urine samples were obtained (at baseline and 6 months post-treatment with anti-rheumatic drugs, including methotrexate, leflunomide, and biologic disease-modifying anti-rheumatic drugs (DMARDs)), 28 (56%) showed a good response as determined by the EULAR response criteria [16]. Interestingly, urinary sCD14 levels in good responders fell significantly from baseline to the 6-month follow-up visit (from 86.1 (57.0–312.1) to 48.7 (19.1–163.6), *p* = 0.015); this did not happen in moderate-to-no responders (from 52.5 (25.7–246.7) to 81.5 (36.6–279.3), *p* = 0.814) (Figure A2C). Moreover, changes in urinary sCD14 levels showed a positive correlation with changes in the DAS28_ESR_ (*rho* = 0.353, *p* = 0.012). However, the area under a receiver operating characteristic (ROC) curve (AUC) analysis (AUC = 0.660, *p* = 0.215) revealed a lack of differentiation between good responders and moderate-to-no responders (Figure A2D).

Overall, urinary sCD14 levels measured by the FREND™-CD14 system showed only modest diagnostic performance for assessing RA activity and for predicting treatment outcome, although results correlated with RA activity and showed excellent analytic performance when compared with those obtained using the conventional ELISA method.

### 3.4. Strong Diagnostic Performance of Serum sCD14 as Measured by the FREND™-CD14 System

In study II, we used a sCD14 test cartridge and the FREND™ system to measure serum sCD14 levels in 62 patients (124 paired samples) (Figure A1). We compared levels with the ESR, CRP, and DAS28_ESR_, which comprises the number of tender joints and swollen joints, a PGA on a visual analogue scale, and ESR values. The result is calculated using the following complex equation [22]:

DAS28_ESR_ = 0.56 √(no. of tender joints) + 0.28 × √(no. of swollen joints) + (0.014 × PGA) + [0.70 × ln(ESR)]

**Figure 2:**
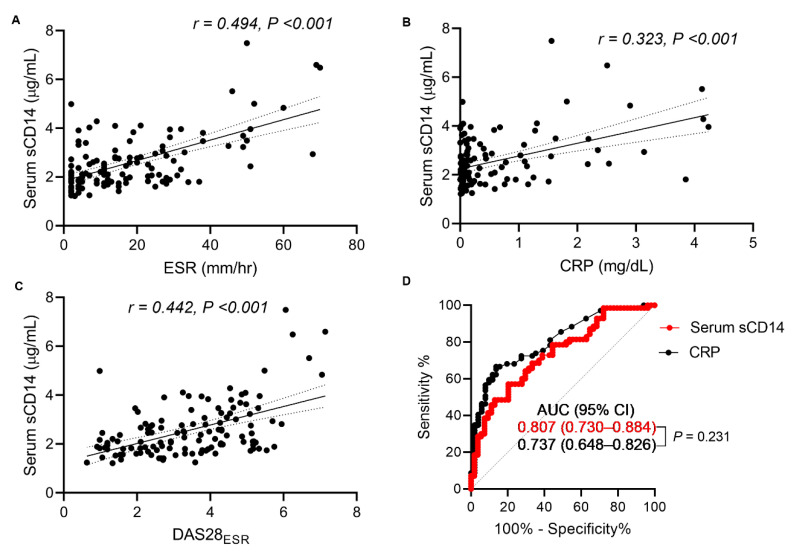
Correlation between serum sCD14 levels (measured by FREND™) with RA disease activity. (**A**–**C**) Spearman’s correlation analysis of the relationship between serum sCD14 levels (measured by the FREND™-CD14 system) and ESR, CRP, and the DAS28ESR. (**D**) Area under a receiver operating characteristic (ROC) curve (AUC) analysis of serum sCD14 to determine its ability to distinguish RA patients with low disease activity (DAS28_ESR_ ≤ 3.2) from those with moderate (3.2 < DAS28_ESR_ ≤ 5.1) or high (DAS28_ESR_ > 5.1) disease activity according to ACR recommendations.

The baseline characteristics of the RA patients in the serum study (study II) are shown in Table A1; the characteristics were similar to those of patients in the urine study (study I). The results showed that, in contrast with urine sCD14 levels, serum sCD14 levels measured by the FREND™-CD14 system correlated with ESR, serum CRP, and the DAS28_ESR_, indicating that they reflect RA disease activity well; the correlation coefficients were 0.494 for ESR (*p* < 0.001), 0.323 for CRP (*p* < 0.001), and 0.442 for the DAS28_ESR_ (*p* < 0.001) (Figure 2A–C). Moreover, serum sCD14 levels measured by FREND™-CD14 differentiated RA patients with low disease activity from those with moderate-to-high disease activity (Figure 2D). The AUC was 0.737 (*p* < 0.001), which was comparable with that for CRP (AUC = 0.807, *p* = 0.231 vs. sCD14) (Figure 2D), confirming that sCD14 measured by FREND™-CD14 is an excellent indicator of RA disease activity.

**Figure 3:**
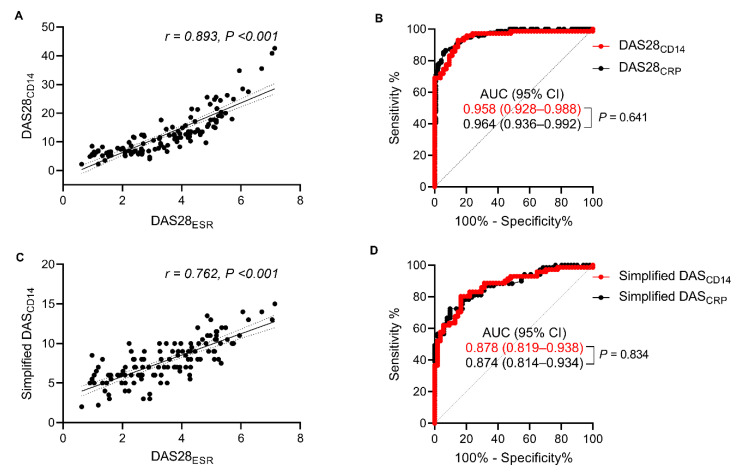
Diagnostic performance of the new composite indices based on CD14 concentrations. (**A**) Correlation between the DAS28_CD14_ and DAS28_ESR_. (**B**) Area under the receiver operating characteristic (ROC) curve (AUC) analysis of the DAS28_CD14_ versus DAS28_CRP_ (as a comparator) for distinguishing low disease activity from moderate/high disease activity, as determined by the DAS28_ESR_. The two compared areas are not significantly different. (**C**) Correlation between the simplified DAS_CD14_ and DAS28_ESR_. The simplified DAS_CD14_ was calculated as follows: “simplified DAS_CD14_ = 0.1 × PGA + sCD14 (μg/mL)”. (**D**) ROC curve analysis of the ability of the simplified DAS_CD14_ to distinguish remission/low DAS28_ESR_ scores from moderate/high DAS28_ESR_ scores. The Simplified DAS_CRP_ was calculated as follows: “simplified DAS_CRP_ = 0.1×PGA + CRP (mg/dL)” for comparison with the simplified DAS_CD14_. The two AUCs are similar.

Based on these findings, we generated a new composite score (called the DAS28_CD14_) comprising four variables: tender and swollen joint count, PGA, and the sCD14 concentration (measured by the FREND™-CD14 system). The DAS28_CD14_ score is calculated as follows: TJS28 + SJC28 + 0.1 × PGA + sCD14 (μg/mL). We then evaluated its diagnostic performance with respect to RA activity. DAS28_CD14_ values correlated strongly with the DAS28_ESR_ (*rho* = 0.893, *p* < 0.001) and discriminated low disease activity from moderate-to-high activity with an AUC of 0.958 (*p* < 0.001), which was comparable with that of DAS28_CRP_ (Figure 3A,B).

It is almost impossible for a patient to accurately assess the number of tender and swollen joints by themselves. Therefore, to assist patient-driven measurement of RA activity at home, we formulated a ‘simplified DAS_CD14_’comprising only two variables (PGA [0–10 cm, 10 = the worst score] and the sCD14 concentration measured by the FREND™-CD14 system. The simplified DAS_CD14_ is calculated as follows: PGA + sCD14 (μg/mL). We then evaluated its diagnostic performance. Surprisingly, similar to DAS28_CD14_, the ‘simplified DAS_CD14_’ scores correlated well with DAS28_ESR_ values (*rho* = 0.762, *p* < 0.001); the correlation coefficient was much higher than that between sCD14 (measured by the FREND™-CD14 system) and the DAS28_ESR_ (Figure 2C and Figure 3C). Moreover, the simplified DAS_CD14_ had an AUC of 0.878 for discriminating low RA disease activity (Figure 3D), indicating that it reflects RA activity well.

Concurrent with this, the calculated κ value for the agreement between the simplified DAS_CD14_ and DAS28_ESR_ for RA activity was 0.53 (95% CI, 0.4–0.65); for this analysis, the thresholds for the simplified DAS_CD14_ with respect to low RA activity and moderate/high RA activity were 7 and 10, respectively (compared with thresholds of 3.2 and 5.1, respectively, for the DAS28_ESR_) [22] (Table 1). The κ value was 0.457 (95% CI, 0.27–0.65) at the initial visit and 0.387 (95% CI, 0.15–0.63) at the 6-month follow-up (Table 1). Weighted κ values showed similar results (Table 1). Additionally, a multivariate model identified the ‘simplified DAS_CD14_’ and ‘DAS28_CD14_’ as being independently associated with the DAS28_ESR_, even after adjusting for age, sex, comorbidities, serological profiles, and medications (overall *R^2^* = 0.658 and 0.808, respectively) (Table A2).

### 3.5. Diagnostic Performance of DAS28_CD14_ and the Simplified DAS_CD14_ for Tracking Treatment Responses

**Figure A3:**
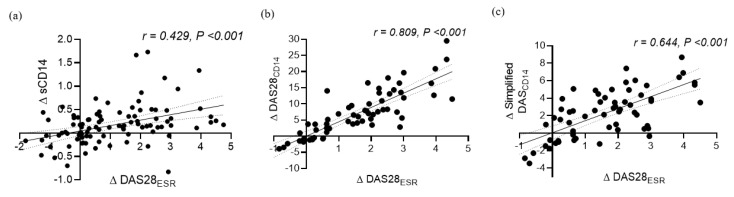
Correlation between changes (Δ) in the DAS28_ESR_ with those in sCD14 (**a**), the DAS28_CD14_ (**b**), and the simplified DAS_CD14_ (**c**), which were calculated as the level at baseline—level at 6 months after treatment with anti-rheumatic drugs.

Finally, we asked whether the DAS28_CD14_ and the simplified DAS_CD14_ (derived from the FREND™-CD14 system) reflect treatment responses to anti-rheumatic drugs after 6 months. As shown in Figure A3a, changes in serum sCD14 (ΔsCD14 = sCD14 concentration at baseline—sCD14 concentration at 6 months after treatment with DMARDs) determined by the FREND™-CD14 system correlated with those identified by the DAS28_ESR_ (ΔDAS28_ESR_) (*rho* = 0.429, *p* < 0.001). Changes in the DAS28_CD14_ (ΔDAS28_CD14_) or simplified DAS_CD14_ (Δsimplified DAS_CD14_) correlated strongly with the ΔDAS28_ESR_ (*rho* = 0.644 and *rho* = 0.850, respectively) (Figure A3b,c), confirming that they are a good representative measure of RA activity.

**Figure 4:**
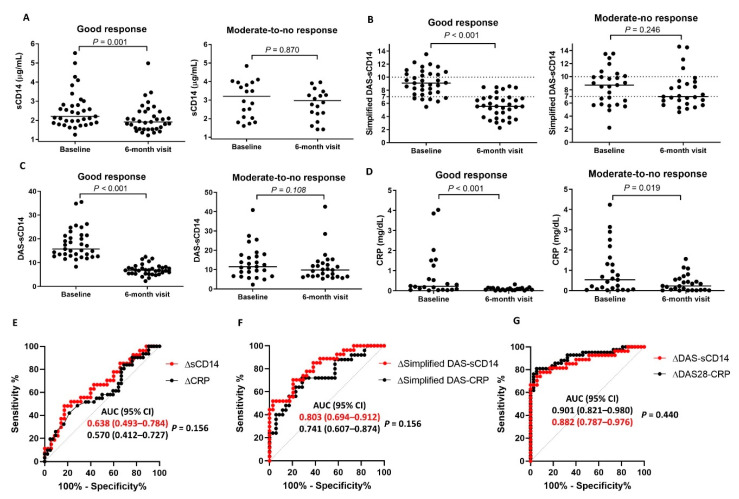
Levels of serum sCD14 (measured by FREND™) DAS28_CD14_, simplified DAS_CD14_, and CRP according to treatment response. (**A**–**C**) The results for serum sCD14 (**A**), DAS28_CD14_ (**B**), simplified DAS_CD14_ (**C**), and CRP (**D**) are shown at baseline and at 6 months post-treatment with anti-rheumatic drugs. Good, moderate, and no response were defined according to European League Against Rheumatism (EULAR) response criteria. (**E**–**G**) The AUC analysis of the ability of ΔsCD14 (**E**), ΔDAS28_CD14_ (**F**), and the Δsimplified DAS_CD14_ (**G**), calculated as the level at baseline to the level at 6 months after treatment with anti-rheumatic drugs, to distinguish a good response from a moderate-to-no response. The AUC of ΔCRP and Δsimplified DAS_CRP_ (=0.1 × PGA + CRP [mg/dL]) are shown for comparison.

Similar to study I, study II showed that 35 patients (56.5%) experienced a good response according to EULAR criteria [16]. In this cohort, serum sCD14 levels, the DAS28_CD14_, and the simplified DAS_CD14_ fell significantly in good responders (n = 35) but showed no change in moderate-to-no responders (n = 27), indicating that these scores may be a good alternative for assessing treatment responses in RA patients (Figure 4A–C). Interestingly, although CRP levels fell considerably in responders (as expected), they also fell in non-responders 6 months after DMARD treatment (Figure 4D); this suggests that CRP levels fall non-specifically, irrespective of therapeutic outcome.

ROC analysis showed that neither ΔsCD14 nor ΔCRP (CRP level at baseline to the CRP level at 6 months after treatment with DMARDs) distinguished between good EULAR treatment responders and moderate-to-no responders (AUC = 0.638 and *p* = 0.121 for ΔsCD14; AUC = 0.570 and *p* = 0.191 for ΔCRP) (Figure 4E). However, ΔDAS28_CD14_ strongly discriminated good EULAR treatment responders from moderate-to-no responders (AUC = 0.882 (*p* < 0.001), which was comparable with the AUC for DAS28_CRP_ (*p* = 0.440)) (Figure 4F). Notably, the Δsimplified DAS_CD14_ also showed a good performance for discriminating good EULAR treatment responders (Figure 4G), suggesting that it may be a promising surrogate parameter that enables us to predict treatment responses without assessing the number of tender and swollen joints.

## 4. Discussion

Recent recommendations for RA treatment endorse the treat-to-target strategy [23,24]. The target is defined as remission or low disease activity, as specified by composite disease activity indices [2]. Although the treat-to-target strategy is the ideal, it is not the case in the real-world [25,26]. Assessing a composite index at every visit is a barrier to implementation of the treat-to-target strategy. A previous study reveals that physicians recorded at least one treat-to-target-related aspect (usually a measure of disease activity) in only 35.7% of visits [27]. Another barrier is doubt about whether disease activity indices invariantly reflect RA activity. Indeed, composite indices can be affected by patient-specific factors, particularly comorbidities such as fibromyalgia [28]. Moreover, clinical measures, including tender and swollen joint counts, exhibit significant inter-assessor and inter-subject variability [29]. Objective measures such as biomarkers may be necessary to complement or support the current indices for RA activity.

Mindful of debate on the current measures for RA activity, we conducted a prospective cohort study to determine whether levels of sCD14, a co-receptor for detection of lipopolysaccharide [12], represents RA activity and treatment responses to DMARDs when measured rapidly using the FREND™-CD14 system. We demonstrated that the new assay was capable of accurately quantifying sCD14 levels in a drop of serum or urine within 5 min. Using this system, we showed that, as a reflection of disease activity and treatment responses in RA, sCD14 levels are comparable with CRP levels, which is consistent with earlier reports by our group and others [10,11,12]. On the basis of this finding, we created a new composite score for RA activity comprising tender and swollen joint counts, serum sCD14 concentrations measured using FREND™, and/or PGA. In particular, the DAS28_CD14_ and simplified DAS_CD14_ scores correlated well with DAS28_ESR_ and exhibited excellent diagnostic performance with respect to good responses to DMARDs treatment at 6 months.

Development of a rapid immune-assay system, such as the FREND™-CD14 system, is not always possible; as it is not compatible with all proteins, and it requires a highly efficient antibody, in addition to the presence of measurable amounts of target protein in bio-fluids. Here, we developed a rapid quantitative immunoassay for sCD14 and demonstrated that sCD14 concentrations in urine and serum are associated with RA activity and treatment responses. Originally, we expected urine to better fit our goal than serum because it is more stable and can be collected at home by patients. Unfortunately, the diagnostic performance of urinary sCD14 measured using the FREND™ was modest when compared with that of serum sCD14. Even though urine is an appropriate bio-fluid for self-monitoring of RA activity, it contains a variety of cellular elements, organic molecules, inorganic crystals, and casts. The variability of urine matrix components, mostly due to differences in the daily intake of fluid and diet, results in less reproducibility and undermines the assay performance of urinary biomarkers [21,30].

Many practicing rheumatologists feel that measurement and documentation of composite disease activity indices at every visit is burdensome. Therefore, a single biomarker that provides an intuitive estimation of RA activity is preferred, although it is hard to capture the full complement of RA processes using a single indicator. Our results demonstrate a significant relationship between DAS28_ESR_ and sCD14 levels measured by the FREND™-CD14 system, DAS28_CD14_, and the simplified DAS_CD14_, suggesting the clinical utility of rapid CD14 and CD14-based composite indices for determining RA activity. Of note, the simplified DAS_CD14_, which can be calculated easily by adding PGA to the sCD14 level, better represented high RA activity than the single sCD14 value. Considering that inflammatory markers such as ESR and CRP do not often correlate with RA activity assessed by joint counts and global assessments [31], we presume that the simplified DAS_CD14_ may compensate for the limitations of a single item (sCD14 and PGA). PGA has advantages in that it is more easily measured than joint counts or acute phase reactants, and it summarizes all aspects of the disease that are important to patients [13,32]. We expect that the new composite index, the simplified DAS_CD14_, is a promising surrogate for assessing RA activity without the need for either tender and swollen joint counts, nor ESR and CRP measurement.

The efforts to find biomarkers for RA that are not affected by treatment or serologic profiles continue [5,33]. Here, we show that sCD14 levels measured by FREND™ were not greatly affected by prednisolone, anti-rheumatic drugs (including methotrexate, hydroxychloroquine, and leflunomide), or some cytokines. Moreover, an analysis of covariance model demonstrated that CD14-based composite indices were independent of treatment modality. Meanwhile, sCD14 levels, DAS28_CD14_, and the simplified DAS_CD14_, but not CRP, showed a good correlation with treatment responses to DMARDs after 6 months, indicating that they represent clinical outcome irrespective of treatment modality. Moreover, the ΔDAS28_CD14_ and Δsimplified DAS_CD14_ scores showed good power for discriminating good responders from moderate-to-no responders (AUC = 0.882 and 0.803, respectively), suggesting that they could predict therapeutic outcomes.

Another unresolved issue is how often we should assess RA activity. Treat-to-target guidelines start with the premise that intensive monitoring yields better outcomes for patients with highly active RA. Here, we demonstrated that we can use a portable machine to measure sCD14 levels in a drop of bio-fluid within 5 min; this machine can be used in either outpatient clinics or at home. Due to its simplicity and immediacy, the new platform will improve point-of-care testing and, eventually, make it easier to implement treat-to-target strategies more frequently in real-world clinical settings. If we develop the FREND™-CD14 system based on a drop of whole blood, it will also be possible to assess RA activity at home (as is the case for blood glucose monitoring). We anticipate that cumulative data from the sCD14 and simplified DAS_CD14_ measures can be shared with patients and caregivers via mobile applications to enable patient-oriented tight control of RA; the data are likely to more accurately reflect RA activity than data derived from a single visit to hospital.

This study has some limitations. First, it comprised a relatively small number of patients from a single center and did not address the association between the simplified DAS_CD14_ data and radiographic progression. Further studies are needed to confirm the diagnostic performance of both sCD14 measured by FREND™ and the simplified DAS_CD14_ score in a broader population of RA patients. Second, we adopted the DAS28_ESR_ as a standard for RA activity as it is the most widely used measure for clinical research. However, it also has disadvantages, including placing higher weight on tender joints than on swollen joints; although rheumatologists consider the latter more important when making treatment decisions [34]. Moreover, if RA dominantly affects the feet (which are not included in the 28 joint count), the score may be misleadingly low. Therefore, validation against other indices such as the Simplified Disease Activity Index (SDAI) is required.

## 5. Conclusions

We have developed a new system, the FREND™-CD14 system, which uses a drop of bio-fluid to measure RA activity within 5 min. Using this system, we demonstrated that CD14 concentrations and CD14-based composite indices reflect RA activity and treatment outcomes. In particular, the simplified DAS_CD14_ may be a promising surrogate for assessing disease activity without the need for tender and swollen joint counts and ESR/CRP measures. It also offers complementary dimension for more tight and frequent monitoring of RA.

## Figures and Tables

**Table 1:** Classification of disease activity (low, moderate, or high) according to the simplified DAS_CD14_ and DAS28_ESR._

	DAS28_ESR_								
	Baseline Visit			6 Month Visit			Combination (Baseline and 6 Month)		
Simplified DAS_CD14_	Low	Moderate	High	Low	Moderate	High	Low	Moderate	High
Low	7	0	0	38	9	0	45	14	0
Moderate	9	21	6	5	6	2	9	27	6
High	0	6	13	0	0	2	0	8	15
κ (95% CI)	0.457 (0.270–0.645)			0.387 (0.151–0.625)			0.525 (0.397–0.652)		
Weighted κ (95% CI)	0.541 (0.375–0.707)			0.476 (0.245–0.707)			0.622 (0.514–0.730)		

## Supplementary Materials

The following are available online at https://www.mdpi.com/2077-0383/9/11/3499/s1 Table S1: Summary of the precision test for soluble CD14 (sCD14) using the FRENDTM system, Table S2: Measurement of sCD14 FREND TM and sCD14 test cartridge and recovery from specimens containing potential interferents using.

## Appendix A

**Table A1:** Patient characteristics.

	Study I	Study II
Number of patients/samples	50/100	62/124
Age, yr (IQR)	55 (46–62)	58 (52–64)
Female, n (%)	44 (88)	52 (84)
ACPA positive, n (%)	39/44 (88.6)	46/50 (92)
RF positive, n (%)	44 (88)	49/61 (80)
Smoker, n (%)	2 (4)	3 (4.8)
Methotrexate, n (%)	71 (71)	80 (64.5)
Leflunomide, n (%)	43 (43)	40 (32.2)
HCQ, n (%)	47 (47)	59 (47.6)
bDMARDs, n (%)	32 (32)	58 (46.8)
TJC	2 (0–5)	2 (0–4)
SJC	1 (0–3)	1 (0–2)
PGA	55 (40–74)	50 (36–70)
ESR, mm/hr	29 (13–45)	14 (5–30)
CRP, mg/dL	0.41 (0.1–1.7)	0.16 (0.05–0.80)
DAS28_ESR_	4.0 (2.8–5.2)	3.4 (2.2–4.6)
Baseline DAS28_ESR_	5.2 (4.5–5.7)	4.4 (3.8–5.2)
Follow-up DAS28_ESR_	2.8 (2.4–3.4)	2.6 (2.0–3.3)

For studies with two samples per patient, sex, age, smoking, serological status (when available), and baseline and follow-up DAS28_ESR_ statistics are based on single individual. Other statistics are based on all samples. ACPA: anti-citrullinated protein antibody, bDMARDs: biologic disease modifying-anti-rheumatic drugs, DAS28: disease activity score for 28 joints, HCQ: hydroxychloroquine, IQR: interquartile range, PGA: patient global assessment, RF: rheumatoid factor, SJC: swollen joint counts, and TJC: tender joint counts.

**Table A2:** Analysis of covariance (ANCOVA) of DAS28_ESR_, adjustment for various covariates, including newly developed CD14-based RA disease activity indices.

		Model 1 *			Model 2 ^†^		
		Beta	SE	*p*	Beta	SE	*p*
Age	Per 1 Year	0.016	0.011	0.167	0.014	0.009	0.118
Sex	Female vs. Male (ref)	0.034	0.326	0.917	0.218	0.244	0.374
Body Mass Index	Per 1 kg/m^2^	0.038	0.038	0.318	0.051	0.028	0.077
Hypertension	Yes vs. No (ref)	−0.389	0.304	0.203	−0.085	0.227	0.710
Diabetes Mellitus	Yes vs. No (ref)	−0.061	0.498	0.903	0.480	0.373	0.202
Rheumatoid Factor Positive	Yes vs. No (ref)	0.411	0.293	0.165	0.351	0.220	0.114
Acpa Positive	Yes vs. No (ref)	0.302	0.412	0.465	0.226	0.309	0.467
Methotrexate	Yes vs. No (ref)	−0.475	0.232	0.044	0.020	0.178	0.912
Leflunomide	Yes vs. No (ref)	0.244	0.230	0.293	0.160	0.173	0.357
Biologic Dmards	Yes vs. No (ref)	−0.353	0.236	0.137	-0.199	0.173	0.357
Simplified Das_cd14_	Per 1 Score	0.453	0.038	<0.001			
Das28_cd14_	Per 1 Score				0.169	0.010	<0.001

* Multivariate ANCOVA was conducted to estimate the coefficients and SEs for the association between DAS28_ESR_ and independent variables. After adjusting for age, sex, body mass index, baseline comorbidities, serologic markers, and medications, the simplified DAS_CD14_ was independently associated with DAS28_ESR_. This model accounted for approximately 66% of the variance in the DAS28_ESR_ (*R^2^* = 0.658). † To avoid multicollinearity with the simplified DAS_CD14_, a separate model was adopted for DAS28_CD14_. This model explained approximately 81% of the variance in the DAS28_ESR_ (*R^2^* = 0.808).

ACPA: anti-citrullinated protein antibody, DMARDs: disease-modifying-anti-rheumatic drugs, SE: standard error.
