# Supplementary material for: Development of Monitoring System for Assessing Rheumatoid Arthritis within 5 Minutes Using a Drop of Bio-Fluids
Source: J Clin Med. 2020 Oct 29;9(11):3499. doi: 10.3390/jcm9113499 (PMC7692111; doi:10.3390/jcm9113499)
Supplement: Supplementary file 1 [file jcm-09-03499-s001.pdf]

Supplemental Data

Development of Monitoring System for Assessing Rheumatoid Arthritis within 5 Minutes using a Drop of Bio-fluids

Supplemental Table 1. Summary of the precision test for soluble CD14 (sCD14) using the FREND^{TM} system

sCD14 (ng/mL)	5		20		100	
	SD	CV (%)	SD	CV (%)	SD	CV (%)
Repeatability	0.54	10.1	1.48	7.3	6.36	6.2
Between Run	0.00	0.0	1.16	5.8	3.03	2.9
Within Day	0.54	10.1	1.88	9.3	7.04	6.9
Between Day	0.12	2.2	0.00	0.0	0.00	0.0
Within Laboratory	0.56	10.3	1.88	9.3	7.04	6.9
Lot-to-Lot	0.34	6.5	1.38	6.7	5.24	5.0

CV, coefficient of variation; SD, standard deviation

Supplemental Table 2. Measurement of sCD14 FREND™ and sCD14 test cartridge and recovery from specimens containing potential interferents using

Interfering substance	Concentration	Recovery (%)
Acetylsalicylic acid	200 μg/mL	91.9
Ascorbic acid	200 μg/mL	98.5
Bilirubin	20 μg/mL	105.1
Glucose	20 μg/mL	98.4
Prednisolone	1 μg/mL	102.9
Methotrexate	1 μg/mL	103.5
Tacrolimus	1 μg/mL	97.4
Leflunomide	1 μg/mL	113.4
Hydroxychloroquine	1 μg/mL	101.1
GM-CSF	1 μg/mL	97.7
IL-4	1 μg/mL	89.2
TNF-α	1 μg/mL	88.6
IL-13	1 μg/mL	104.3

IL, interleukin; GM-CSF, granulocyte-macrophage colony-stimulating factor; TNF-α, tumor necrosis factor-α.
